# Preoperative systemic immune-inflammation index-based nomogram for lung carcinoma following microwave ablation -a real world single center study

**DOI:** 10.3389/fonc.2024.1305262

**Published:** 2024-03-20

**Authors:** Jing Wang, Song-ping Cui, Qing Zhao, Ya Gao, Ying Ji, Yi Liu, Jin-bai Miao, Yi-li Fu, Bin Hu

**Affiliations:** Department of Thoracic Surgery, Beijing Institute of Respiratory Medicine and Beijing Chao-Yang Hospital, Capital Medical University, Beijing, China

**Keywords:** lung carcinoma, microwave ablation, biomarkers, systemic inflammatory response index, prognosis

## Abstract

**Background:**

The preoperative inflammatory condition significantly influences the prognosis of malignancies. We aimed to investigate the potential significance of preoperative inflammatory biomarkers in forecasting the long-term results of lung carcinoma after microwave ablation (MWA).

**Method:**

This study included patients who received MWA treatment for lung carcinoma from Jan. 2012 to Dec. 2020. We collected demographic, clinical, laboratory, and outcome information. To assess the predictive capacity of inflammatory biomarkers, we utilized the area under the receiver operating characteristic curve (AUC-ROC) and assessed the predictive potential of inflammatory biomarkers in forecasting outcomes through both univariate and multivariate Cox proportional hazard analyses.

**Results:**

A total of 354 individuals underwent MWA treatment, of which 265 cases were included in this study, whose average age was 69.1 ± 9.7 years. The AUC values for the Systemic Inflammatory Response Index (SIRI) to overall survival (OS) and disease-free survival (DFS) were 0.796 and 0.716, respectively. The Cox proportional hazards model demonstrated a significant independent association between a high SIRI and a decreased overall survival (hazard ratio [HR]=2.583, P<0.001). Furthermore, a high SIRI independently correlated with a lower DFS (HR=2.391, P<0.001). We developed nomograms utilizing various independent factors to forecast the extended prognosis of patients. These nomograms exhibited AUC of 0.900, 0.849, and 0.862 for predicting 1-year, 3-year, and 5-year OS, respectively. Additionally, the AUC values for predicting 1-year, 3-year, and 5-year DFS were 0.851, 0.873, and 0.883, respectively.

**Conclusion:**

SIRI has shown promise as a valuable long-term prognostic indicator for forecasting the outcomes of lung carcinoma patients following MWA.

## 1 Introduction

Incidence and mortality rates of pulmonary carcinoma account for 17.1% and 21.7% of all malignant tumors, respectively, making it a major public health concern that significantly threatens human well-being (1). The treatment of lung carcinoma, both primary and metastatic, has undergone substantial diversification over the past two decades. New therapeutic technologies offer a range of choices, from traditional open surgical resection and video-assisted thoracoscopic surgery (VATS) to more recent highly efficient radiation therapy methods, exemplified by stereotactic body radiation therapy (SBRT) and image-guided thermal ablation treatments such as radiofrequency ablation (RFA), microwave ablation (MWA), and cryoablation. Multitude of techniques and their constant improvement raise the question of which approach is most beneficial for optimal patient outcome. Although SBRT and thermal ablation treatments exhibit lower control rates compared to surgical resection in the overall population of lung carcinoma patients, their primary advantages lie in reducing invasiveness and their impact on respiratory function. Additionally, they demonstrate prognosis comparable to surgery in certain early-stage non-small cell lung carcinoma (NSCLC) (2, 3).

Hence, they offer a potential curative option for patients with early-stage NSCLC or limited metastasis for whom surgical intervention is medically infeasible. Over the past five years, thermal ablation, particularly MWA, has made significant strides in the treatment of lung tumors. MWA holds theoretical advantages over RFA, as it can generate a larger ablation zone in a shorter duration. Initially, this local treatment technique was primarily employed for lung carcinoma patients who were ineligible for surgery or unsuitable candidates for surgical intervention. However, with advancements in technology and the accumulation of clinical experience, its indications have expanded to include the treatment of early-stage multiple primary lung carcinomas (MPLC), especially in cases where tumors cannot be surgically resected through conventional means or when patients are averse to surgery (4). Multiple studies have indicated that MWA can achieve high rates of local control, particularly for smaller ground-glass opacities (GGO) or ground-glass nodules (GGN) (5). The use of next-generation radiofrequency ablation devices and advanced medical imaging techniques such as CT and electromagnetic navigation technology has enhanced the precision of MWA procedures, ensuring more accurate tumor localization and treatment (6).

Although some literature reports that for early-stage NSCLC patients, the survival rates after MWA treatment are comparable to traditional surgical approaches, with a lower incidence of complications (7), there is limited documentation regarding post-MWA recurrence and prognosis for the overall lung carcinoma patient population, therefore, effective biomarkers and accurate prediction of the risk of tumor recurrence after MWA for lung carcinoma and timely intervention are particularly important for improving the disease free survival (DFS) and overall survival (OS).

The deviation from homeostasis is resolved through an intensified immune activation process, often referred to as inflammation. The malignant transformation of cells represents such a non-physiological state, demanding a robust host response. The current consensus suggests a close association between chronic inflammation and the development of tumors. Thus far, it has been postulated that many cancers are either caused by chronic inflammation or induced by inflammatory reactions, making inflammation a characteristic feature of cancer (8). MWA releases a microwave thermal field that induces rapid rotational motion and friction of surrounding molecules, raising temperatures to promote coagulative necrosis in both the tumor and adjacent tissues. Simultaneously, the release of numerous cellular debris and inflammatory factors resulting from necrosis further stimulates the occurrence of tumor immune responses (9). In the meantime, inflammatory and immune responses related to tumors are recognized as crucial factors in the initiation, progression, angiogenesis, and metastasis of tumors (10, 11). These include several inflammatory and immune scores, such as neutrophil lymphocyte ratio (NLR), platelet lymphocyte ratio (PLR) and monocyte-to-lymphocyte ratio (MLR) (12). A recently introduced systemic inflammatory response index (SIRI), calculated from absolute lymphocyte, monocyte, and neutrophil counts in peripheral blood, has emerged as a promising prognostic tool for various cancers, including NSCLC (13, 14). Numerous investigations have provided evidence that increased SIRI values are linked to unfavorable outcomes among NSCLC patients following surgical resection (15, 16). However, the threshold for SIRI values vary widely across the studies (17, 18) and the prognostic value of preoperative systemic immune-inflammation index in patients with early-stage pulmonary cancer following MWA remains unclear.

This real-world study was conducted to investigate the prognostic value of SIRI in patients with lung carcinoma after MWA treatment. Novel preoperative SIRI-based nomograms were developed to predict the probability of MWA in these patients to help screen high-risk patients and formulating adjuvant therapeutic and individualized strategies.

## 2 Materials and methods

### 2.1 Study population

This study included patients who received MWA treatment for lung carcinoma at our hospital from January 2012 to December 2020.

Inclusion Criteria: (1) patients >18 years old; (2) the pathological results confirm NSCLC; (3) not suitable for curative surgery or patient refuses curative surgery; (4) no signs of metastasis; (5) ECOG score of 0-1; (6) no preoperative neoadjuvant therapy; (7) no history of radiation therapy; (8) informed consent from patients and their families regarding the surgical procedure and research protocol.

Exclusion criteria: (1) consideration of metastatic lung carcinoma or lymph node metastasis; (2) concurrent active bacterial or fungal infections; (3) deemed unsuitable for inclusion by the research team after evaluation; (4) incomplete follow-up information.

### 2.2 Data collection and follow-up

We collected the following data using an electronic medical record system: patient baseline information (age, gender, smoking history, and family history, etc.), laboratory test results (complete blood count, etc.), surgical-related data (surgery time, MWA power, intraoperative bleeding volume, chest drainage volume, pneumothorax, etc.), complications (pneumothorax, hemothorax, pulmonary hemorrhage) and pathological results (pathological type, degree of differentiation, etc.). All patients who were enrolled had venous blood samples collected within 24 hours of admission and underwent a complete blood count analysis. NLR was defined as the ratio of neutrophils to lymphocytes, while PLR was defined as the ratio of platelets to lymphocytes. SII was defined as the neutrophil counts multiplied by the platelet counts, then divided by the lymphocyte counts. SIRI was defined as the monocyte counts multiplied by the neutrophil counts divided by the lymphocyte counts.

We maintained continuous patient follow-up through telephone calls, outpatient visits, or inpatient observation until December 2022 or until the occurrence of death. Follow-up of discharged patients at 6 months, 1 year, and 2 years post-surgery to assess the occurrence of short-term and long-term complications, as well as their survival status, etc. The overall survival (OS) was defined as the time from MWA to death or the last observation, while disease-free survival (DFS) was defined as the time from MWA to recurrence.

### 2.3 MWA procedure

Computerized Tomography (CT) and magnetic navigation system were employed to guide the MWA procedure. This study is real-world research, and there are no specific eligibility criteria for patient inclusion. The choice between CT or magnetic navigation system-guided surgery can be made through communication between patients and physicians. MWA employs the MTC-3C microwave ablation system (developed by Zhongyuan Medical Device Research Center) with a frequency of 2450 ± 50 MHz and an adjustable continuous wave output power range from 0 to 100W. The effective length of the microwave antenna is 100-180mm, with an outer diameter of 14-20G. The effective ablation range of MWA is 3.5 cm * 3 cm, with an output of 60~80W/6~8 min. In this study, the high-power group is defined as having an output power of 80W, while the low-power group is defined as having an output power of 60W. The surgery is performed by the chief physician, who has extensive experience in tumor ablation to ensure consistency in the quality of the surgery. The radiological manifestation of a successful surgery is that the lesions after ablation is 5-10mm larger than the target lesion.

### 2.4 Statistical analysis

Continuous variables were compared between groups using either the Student’s t-test or the Mann-Whitney U test. Categorical variables were assessed for group comparisons using either Pearson’s chi-square test or Fisher’s exact test. We utilized Kaplan-Meier curves in conjunction with the log-rank test for survival analysis. The receiver operating characteristics (ROC) curve was used to determine the optimal cut-off value. Cox regression models were used to identify independent prognostic factors associated with OS and DFS. Variables were considered for inclusion in the multivariate analysis if their P<0.2 in the univariate analysis. Utilizing independent risk factors identified in Cox analysis, we developed nomograms to predict the long-term prognosis of MWA patients. Statistical significance was defined as two-sided P<0.05. All statistical analyses were performed using GraphPad Prism (version 8.0), SPSS (version 26.0) and R software (version 4.3.1).

## 3 Results

### 3.1 Patient’s basic information

**Figure 1:**
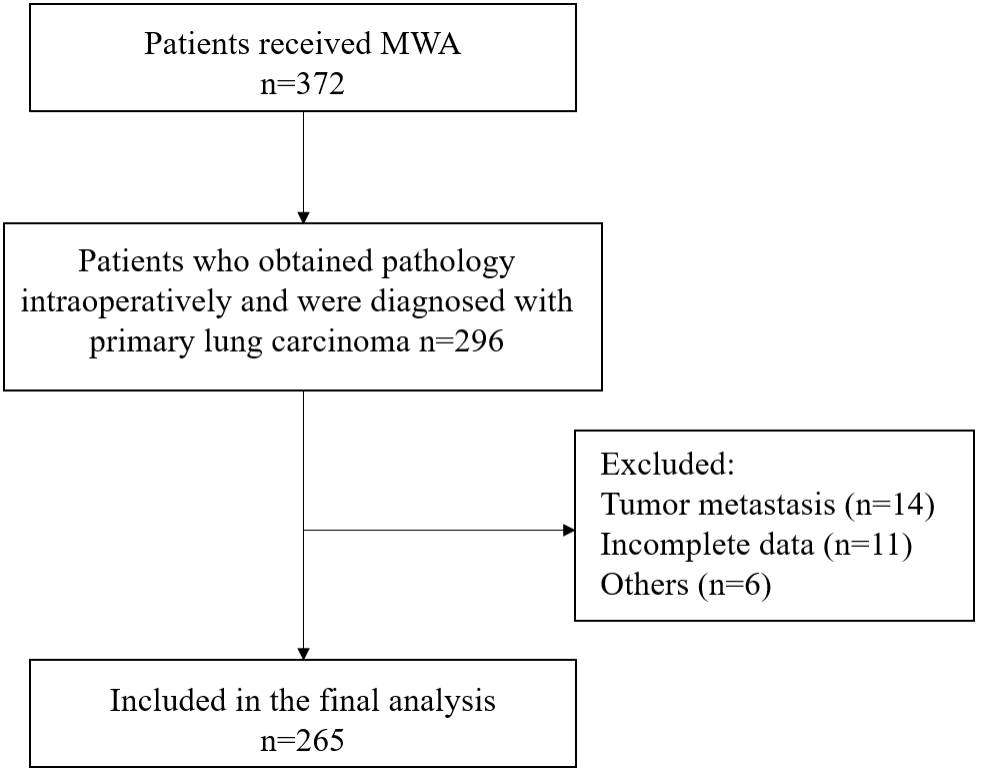
Flowchart depicting the composition of the study group. MWA, microwave ablation.

This study included a total of 265 lung carcinoma patients who underwent MWA (**Figure 1**). The average age of all enrolled patients was 69.1 ± 9.7 years, with 177 males and 88 females. The median duration of follow-up was 43.8 months. The patient’s comorbidity, tumor-related information, and laboratory test results were as shown in **Table 1**.

**Table 1:** Characteristics of the entire cohort in terms of demographics and baseline data.

Variables	Total (n=265)	Low SIRI group (n=188)	High SIRI group (n=77)	P
Age	69.1 ± 9.7	68.5 ± 9.4	70.7 ± 10.1	0.103
Gender (male)	177 (66.8)	115 (61.2)	62 (80.5)	<0.001
Comorbidity				
Hypertension	68 (25.7)	44 (23.4)	24 (31.2)	0.189
Diabetes	35 (13.2)	22 (11.7)	13 (16.9)	0.258
Coronary heart disease	15 (5.7)	9 (4.8)	6 (7.8)	0.336
Smoking	50 (18.9)	32 (17.0)	18 (23.4)	0.230
Tumor diameter	2.0 ± 1.0	1.8 ± 1.0	2.4 ± 1.0	<0.001
MWA power (High)	73 (27.5)	53 (28.2)	20 (26.0)	<0.001
Complication	92 (34.7)	61 (32.4)	31 (40.3)	<0.001
Tumor differentiation				<0.001
Poor	33 (12.5)	12 (6.4)	21 (27.3)	
Moderate and well	232 (87.5)	176 (93.6)	56 (72.7)	
Gene mutation	66 (24.9)	51 (27.1)	15 (19.5)	<0.001
Neutrophil (×10^9^/L)	4.0 ± 1.9	3.2 ± 0.9	5.9 ± 2.2	<0.001
Lymphocyte (×10^9^/L)	1.7 ± 0.6	1.8 ± 0.5	1.3 ± 0.6	<0.001
Monocyte (×10^9^/L)	0.5 ± 0.2	0.4 ± 0.1	0.5 ± 0.2	<0.001
Platelet (×10^9^/L)	207.3 ± 58.8	206.0 ± 54.1	207.9 ± 66.1	0.810

MWA, microwave ablation.

We applied the ROC curve to explore the correlation between preoperative inflammatory biomarkers and long-term prognosis in lung carcinoma patients after MWA. The results showed that SIRI had the best value for predicting long-term prognosis (**Supplementary Figure 1**). For predicting postoperative OS and DFS, the optimal cutoff values for SIRI were 1.24 and 1.15, respectively (area under the curve [AUC] = 0.796, 0.716, respectively). Based on the OS corresponding cut-off value, divide all patients into two groups for a comparison of basic information. The results indicated that there were statistically significant differences between the two groups in terms of gender, tumor diameter, MWA power, complication, tumor differentiation, gene mutation, neutrophil count, lymphocyte count, monocyte count, etc. (**Table 1**).

### 3.2 SIRI and long-term prognosis

We applied Cox regression analysis to explore the factors affecting the long-term prognosis of lung carcinoma patients who underwent MWA, and the results were shown in **Tables 2** and **3**. Because there was multicollinearity among neutrophil count, lymphocyte count, monocyte count, and SIRI, and SIRI was more systematic and comprehensive compared to the other three, we have therefore only included SIRI in the multivariate analysis.

**Table 2:** Cox regression examination investigating the impact of clinicopathological variables on patients’ overall survival.

Variables	Univariable Cox regression		Multivariable Cox regression	
	HR (95% CI)	P	HR (95% CI)	P
Age	0.998 (0.977-1.019)	0.844		
Gender (male)	1.213 (0.983-1.523)	0.423		
Tumor diameter	2.122 (1.811-2.487)	<0.001	2.271 (1.865-2.765)	<0.001
MWA power (High)	0.600 (0.369-0.977)	0.040	0.510 (0.306-0.849)	0.010
Complication	1.623 (1.080-2.439)	0.020	1.078 (0.695-1.672)	0.738
Tumor differentiation (Poor)	7.226 (4.496-11.614)	<0.001	3.983 (2.418-6.563)	<0.001
Gene mutation				
No mutations	ref			
EGFR mutations	0.115 (0.042-0.314)	<0.001	0.151 (0.055-0.3418)	<0.001
KRAS mutations	0.453 (0.142-1.446)	0.181	0.269 (0.082-0.885)	0.031
Neutrophil	1.409 (1.277-1.554)	<0.001		
Lymphocyte	0.581 (0.411-0.821)	0.002		
Monocyte	5.485 (1.819-16.537)	0.003		
Platelet	1.002 (0.999-1.005)	0.276		
SIRI (High)	2.753 (1.832-4.137)	<0.001	2.583 (1.675-3.983)	<0.001

MWA, microwave ablation, EGFR, Epidermal Growth Factor Receptor, KRAS, Kirstenratsarcomaviraloncogenehomolog, SIRI, the ratio of monocyte multiply neutrophil to lymphocyte.

**Table 3:** Cox regression examination investigating the impact of clinicopathological variables on patients’ disease-free survival.

Variables	Univariable Cox regression		Multivariable Cox regression	
	HR (95% CI)	P	HR (95% CI)	P
Age	0.990 (0.968-1.012)	0.359		
Gender (male)	1.067 (0.589-1.934)	0.831		
Tumor diameter	2.1647 (1.848-2.495)	<0.001	2.214 (1.859-2.637)	<0.001
MWA power (High)	0.670 (0.423-1.062)	0.088	0.500 (0.309-0.810)	0.005
Complication	1.129 (0.730-1.746)	0.585		
Tumor differentiation (Poor)	6.811 (4.295-10.802)	<0.001	3.604 (2.201-5.901)	<0.001
Gene mutation				
No mutations	ref			
EGFR mutations	0.112 (0.041-0.306)	<0.001	0.144 (0.052-0.395)	<0.001
KRAS mutations	0.754 (0.277-2.055)	0.581	0.554 (0.202-1.521)	0.252
Neutrophil	1.325 (1.167-1.505)	<0.001		
Lymphocyte	0.669 (0.486-0.920)	0.014		
Monocyte	1.491 (0.344-6.463)	0.593		
Platelet	1.002 (0.999-1.005)	0.283		
SIRI (High)	2.878 (1.941-4.266)	<0.001	2.391 (1.573-3.635)	<0.001

MWA, microwave ablation, EGFR, Epidermal Growth Factor Receptor, KRAS, Kirstenratsarcomaviraloncogenehomolog, SIRI, the ratio of monocyte multiply neutrophil to lymphocyte.

Multivariable cox regression analysis showed that tumor diameter, MWA power (High), tumor differentiation (Poor), EGFR mutations, and SIRI (High) were independent related factors for OS (hazard ratio [HR] 2.271, P<0.001; HR 0.510, P=0.010; HR 3.983, P<0.001; HR 0.151, P<0.001 and HR 2.583, P<0.001, respectively; **Table 2**). Tumor diameter, MWA power (High), tumor differentiation (Poor), EGFR mutations, and SIRI (High) were independent related factors for DFS (HR 2.214, P<0.001; HR 0.500, P=0.005; HR 3.604, P<0.001; HR 0.144, P<0.001 and HR 2.391, P<0.001, respectively; **Table 3**).

**Figure 2:**
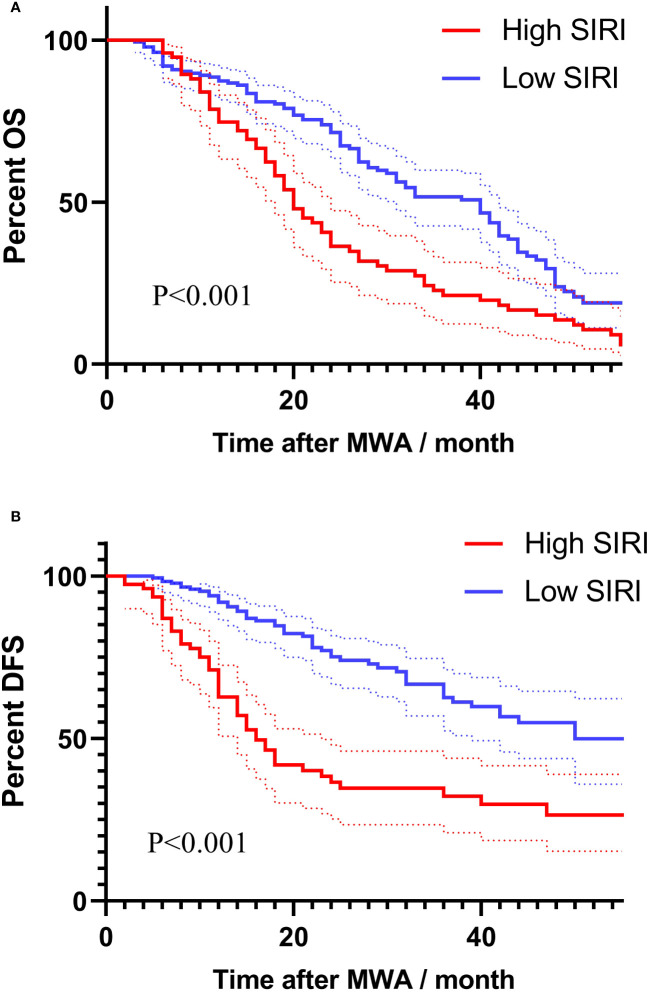
Survival analysis using Kaplan-Meier curves based on SIRI for long-term prognosis: **(A)** Kaplan-Meier survival curves based on SIRI for assessing overall survival. **(B)** Kaplan-Meier survival curves based on SIRI for assessing disease-free survival.

**Figures 2A, B** showed the Kaplan-Meier survival analysis correlating SIRI with the long-term prognosis of lung carcinoma patients receiving MWA. The long-term mortality and tumor recurrence rates of the SIRI (high) group were significantly higher (log-rank test, P<0.001, P<0.001, respectively).

### 3.3 Construct nomograms based on independent related factors

**Figure 3:**
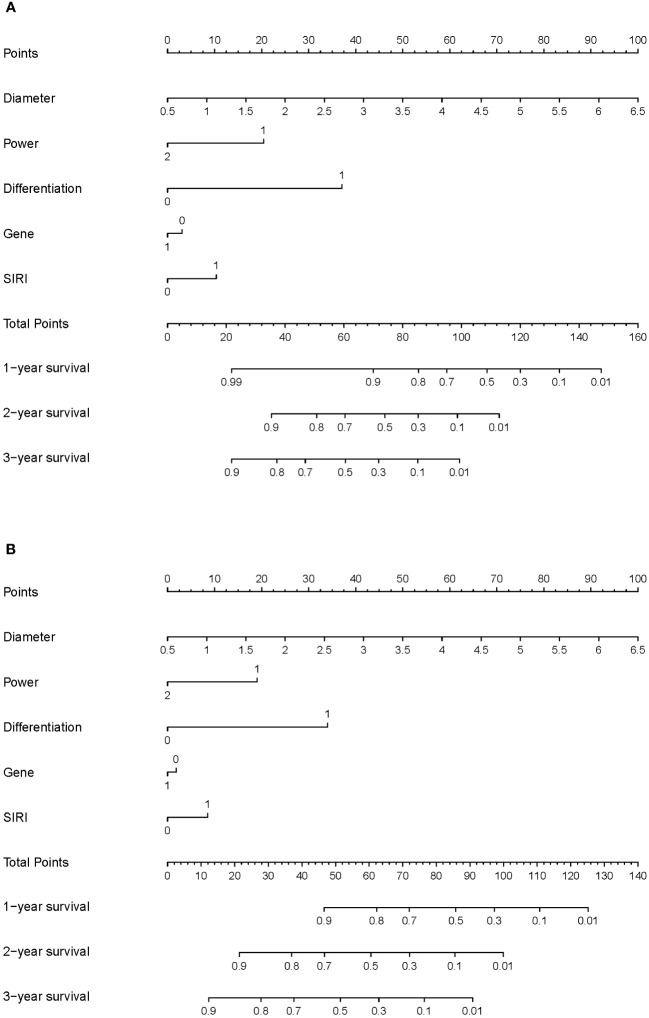
Nomogram for the long-term prognosis of lung cancer patients undergoing MWA: **(A)** Nomogram based on independent related factors for assessing overall survival. **(B)** Nomogram based on independent related factors for assessing disease-free survival. In “Power”, “1” represents the low power group, and “2” represents the high power group. In “Differentiation”, “0” represents the well differentiation group, and “1” represents the poor differentiation group. In “Gene”, “0” represents no gene mutation group and ALK mutation, and “1” represents EGFR mutation group. In “SIRI”, “0” represents the low SIRI group, and “1” represents the high SIRI group.

We constructed nomograms to predict the long-term prognosis of lung carcinoma patients undergoing MWA based on independent factors identified through multivariate Cox regression analysis (**Figures 3A, B**). In the nomograms, each factor is assigned a specific score, and then the cumulative overall risk score is calculated based on these scores. Afterward, it is possible to intuitively predict the long-term prognosis of each patient after MWA by drawing a straight line downward.

**Figure 4:**
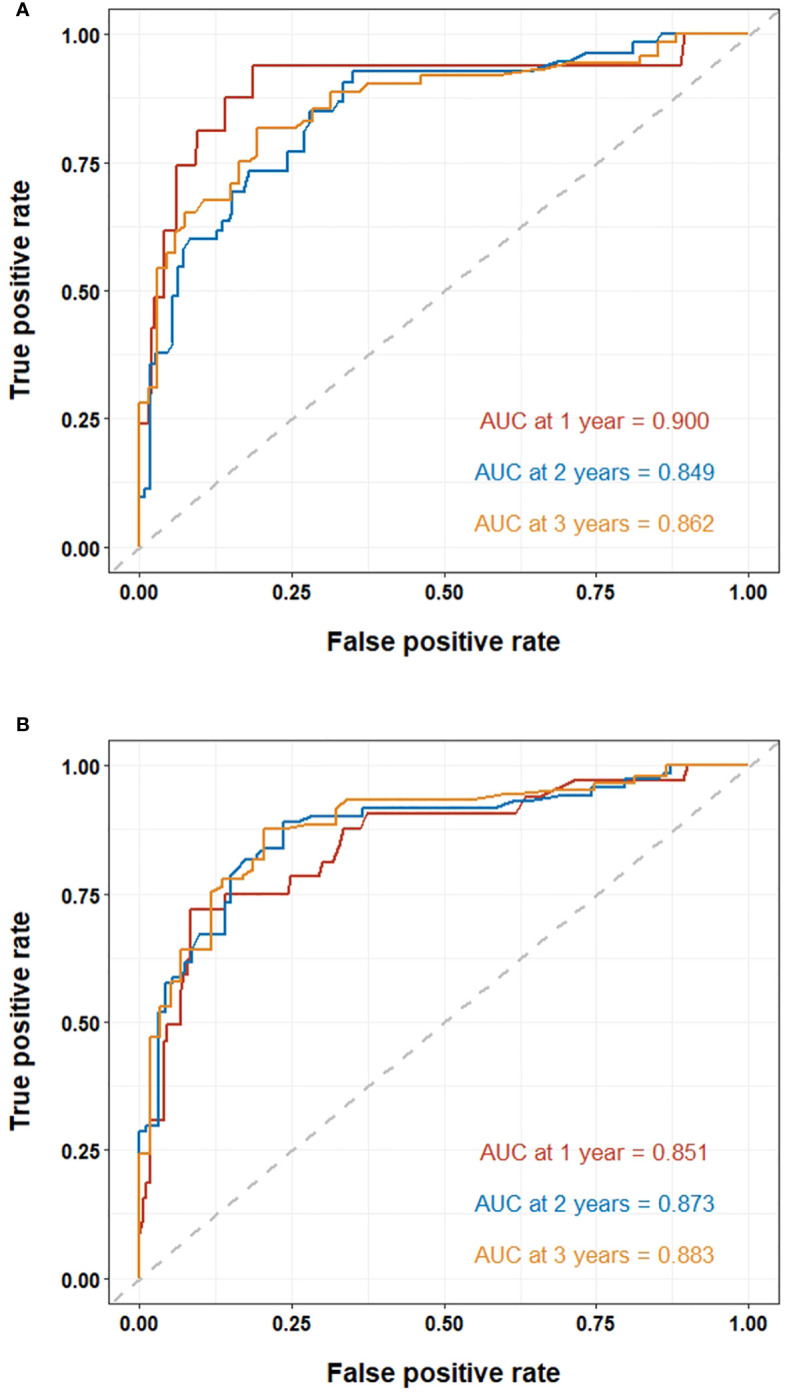
ROC curve of the nomogram for lung cancer patients undergoing MWA: **(A)** ROC curves of the nomogram for 1, 2, and 3-year overall survival. **(B)** ROC curves of the nomogram for 1, 2, and 3-year disease-free survival.

The ROC curves were used to evaluate the predictive value of nomograms for the long-term prognosis of lung carcinoma patients undergoing MWA surgery (**Figures 4A, B**). In OS, the areas under the curves (AUC) corresponding to 1, 2, and 3 years were 0.900, 0.849, and 0.862, respectively. As for DFS, the AUC corresponding to 1, 2, and 3 years were 0.851, 0.873, and 0.883, respectively.

## 4 Discussion

In this real-world study, the prognostic data of 265 pulmonary carcinoma patients following MWA in our center were retrospectively analyzed. SIRI was validated to be a novel and independent factor for predicting DFS and OS, outperforming SII, NLR, PLR and other inflammatory and immune scores. Furthermore, Our study is the first to explore the potential of preoperative SIRI in predicting survival among patients with lung carcinoma treated with MWA worldwide.

The role of chronic inflammation as a carcinogenic factor can be substantiated through various inflammatory conditions that are associated with an increased risk of cancer. The common underlying mechanism involves carcinogenesis through direct mutations or the activation of cellular cytokine responses. Prior research has traditionally concentrated on the impact of the inflammatory process on malignant cells, with a focus on the role of genomic instability and mutations as fundamental concepts in the mechanisms underlying cancer development and pathogenesis (19). MWA, as an emerging interventional therapeutic approach, typically operates at two power frequencies, 915 MHz or 2450 MHz. Under the influence of the microwave electromagnetic field, polar molecules within tumor tissue, such as water and protein molecules, undergo extremely rapid vibrations, leading to mutual collisions and friction between molecules, which resulting in the rapid generation of temperatures ranging from 60 to 150 degrees Celsius within a short timeframe, causing coagulative necrosis of cells. Alongside inducing localized inflammatory changes, the coagulated scar tissue and necrotic tissue may also potentially trigger direct mutagenic effects. Inflammatory cytokines released by immune cells, stromal cells, and tumor cells, activated through nuclear factor-κB and signal transduction and transcription activation factors (STAT), stimulate malignant cell survival and proliferation pathways. Reactive oxygen species (ROS) originating from bone marrow cells can initiate carcinogenic and invasive behaviors (20).

In recent years, the concept of non-mutational epigenetic rearrangements that influence tumor growth and immune regulation has gained increasing attention. It’s noteworthy that a single inflammatory event can drive sustained genetic and epigenetic changes, referred to as epithelial memory, subsequently triggering malignant progression of epithelial cells. In such instances, the continuous adaptive mutations of oncogenes are considered a physiological mechanism to limit tissue damage caused by inflammation. Despite the primary tumor being cleared, cancer patients may still experience recurrence due to dormant cells at distant sites that are clinically undetectable (21).

The role of immune-inflammatory responses and associated cells in the development of tumors is gradually being uncovered. The potential mechanisms underlying the effects of different inflammatory biomarkers on prognosis are unclear; however, they are possibly related to changes in the tumor immune microenvironment. Moreover, tumor immune microenvironment changes are closely associated with inflammatory and immune cell distribution in the peripheral blood (22). As previously reported, biomarkers such as NLR and PLR have prognostic implications in various malignant tumors. Lymphocyte count is closely linked to acquired immunity, and its decrease is advantageous for tumor evasion of immune surveillance, playing a crucial role in the tumor defense system. lymphocytes play a crucial role in cancer therapy, and infiltration by lymphocytes stimulates the production of more pro-inflammatory factors, which promote cytotoxic cell death and inhibit tumor cell proliferation and migration (23, 24). On the contrary, neutrophils not only secrete inflammatory mediators to promote tumor proliferation and metastasis, but also enhance the adhesion ability of distant tumor cells. Elevated neutrophil levels in the bloodstream, along with the heightened expression of cancer-promoting and blood vessel growth-stimulating molecules like vascular endothelial growth factor (VEGF), nuclear factor kappa-B (NF-κB), C-X-C motif chemokine ligand 8 (CXCL8), granulocyte colony-stimulating factor (G-CSF), and transforming growth factor-β1 (TGF-β1), contribute to the creation of a conducive environment for tumor growth (25–27). Platelets contribute to the distant metastasis of tumors by promoting the epithelial-mesenchymal transition of tumor cells, safeguarding their migration to distant organs (28). Meanwhile, increased levels of circulating monocytes have demonstrated an ability to forecast poorer patient survival across various tumor models. Monocytes were observed transitioning into tumor-associated macrophages (29), secreting factors such as TNF-α and VEGF to facilitate both tumor expansion and angiogenesis. Additionally, they hinder the immune response against tumors *in vivo*. These monocytes have also been proven to encourage the movement of tumor cells by releasing enzymes that break down the extracellular matrix (30). In combination, these diverse types of white blood cells collectively constitute the immune milieu, exerting a substantial impact on the therapeutic efficacy of treatments. The Systemic Immune-Inflammation Index (SIRI), calculated based on lymphocyte, neutrophil, and monocyte levels, has demonstrated its ability to predict prognosis for malignancies (31–33). However, SIRI remains relatively unexplored in lung carcinoma patients undergoing MWA treatment. The findings of this study distinctly established a direct correlation between elevated SIRI values and unfavorable long-term outcomes within this particular population. In order to obtain low SIRI, additional neoadjuvant or adjuvant therapy should be performed in patients with MWA.

In recent years, as research into genetic mutations in lung carcinoma has advanced, genetic testing for patients has gained significant value in guiding treatment and predicting prognosis. Common genetic mutations in lung carcinoma include EGFR, KRAS, EML4-ALK, Ros1, and c-MET, among others. Personalized targeted therapies directed at specific mutation sites have become a first-line treatment option for patients with these genetic mutations. Among these, EGFR, being one of the more prevalent mutation genes in NSCLC patients, has seen a continuous evolution of EGFR tyrosine kinase inhibitors (TKIs), significantly improving the survival time of EGFR-positive patients. Since the ADJUVANT study first confirmed the significant prognostic benefit of EGFR-TKI application in postoperative EGFR-positive NSCLC patients (34), multiple subsequent clinical studies have similarly affirmed the positive impact of EGFR-TKIs on patient prognosis, significantly extending both DFS and OS compared to traditional chemotherapy. Guidelines have consequently incorporated targeted therapy as the preferred treatment option for postoperative adjuvant gene mutation-positive patients. Based on our findings, the prognosis of patients with identified gene mutations after MWA treatment was notably better than that of patients without gene mutations, with EGFR mutation-positive patients demonstrating superior outcomes compared to KRAS mutation-positive patients. This outcome is likely attributed to gene mutation-positive patients subsequently receiving targeted drug therapy. Third-generation EGFR-TKI drugs have shown significant efficacy in resistant patients with the T790M mutation, expanding the range of available drugs for EGFR mutation-positive patients and leading to overall improved prognosis. However, adjuvant targeted therapy may not be the sole reason for the extended prognosis in gene mutation-positive patients. A key distinction between our study and others lies in the fact that we examined the post-treatment prognosis of patients with identified gene mutations following MWA treatment, whereas most other studies primarily included postoperative patients. Cell experiments have revealed that the thermal effect increases the levels of the T790M mutation within cells, potentially offering resistant EGFR-TKI patients a longer duration of drug efficacy (35). Xu et al. found that patients with limited progression of TKI resistance significantly benefited from the addition of local ablation therapy (36). Clinical research by Ni et al. indicated that when patients who had experienced EGFR-TKI progression underwent MWA, reusing the same TKI drugs significantly extended both OS and PFS (37). Hence, microwave ablation therapy may prolong the prognosis of gene mutation-positive patients by enhancing the effectiveness of targeted treatments.

Furthermore, our research revealed another significant factor affecting the prognosis of MWA patients is the size of the tumor. Clinically, the maximum diameter of the primary tumor plays a pivotal role in tumor TNM staging and has been affirmed as an independent prognostic factor in several studies concerning lung carcinoma (38, 39). Simultaneously, larger-diameter tumors exhibit a significantly higher rate of recurrence after thermal ablation. Lee et al. found that among 30 lung carcinoma patients undergoing RFA, tumors with a diameter smaller than 3 cm achieved complete necrosis in 100% of cases, whereas this proportion dropped to only 23% in larger one (40). This finding aligns with our study results. In our predictive model, we believe that larger tumor size, which is inherently more invasive, and incomplete ablation due to excessive volume, are both associated with poorer postoperative survival in patients. This observation also provides a solid explanation for the comparison of the prognosis of lung carcinoma patients treated with different power levels of MWA in our other real-world study. Moreover, the degree of tumor differentiation is a crucial factor in assessing tumor malignancy. Poorly-differentiated tumors exhibit greater cellular heterogeneity, with significant differences from normal tissue, and are more prone to local necrosis within the tumor. Sun’s team found that among postoperative NSCLC patients, those with poorly differentiated cancer had a higher Hazard Ratio (HR), indicating a greater risk of recurrence compared to other patients (41). In our study, we conducted the first statistical analysis of the relationship between tumor differentiation and post-MWA prognosis in lung carcinoma patients. The conclusions suggest that the OS of poorly-differentiated group was significantly lower than that of highly-differentiated patients, emphasizing that tumor differentiation should also be considered a risk factor for post-MWA prognosis in lung carcinoma.

It is also noteworthy that in pathological classification, NSCLC accounts for approximately 85% of all lung carcinoma patients, among which the prognostic differences and reasons between lung adenocarcinoma (LUAD) and squamous cell carcinoma (LUSC) have not reached a consistent conclusion in multiple clinical studies. Our real-world study included post-MWA lung carcinoma patients of different pathological types, which is the first reported instance globally. Based on our follow-up data, the short-term OS of LUAD patients was significantly better than that of LUSC patients. This aligns with the findings of the Kawase’s team, although their study attributes this phenomenon not to the histological differences between LUAD and LUSC but to the more significant comorbidity effects in LUSC patients during surgical resection (42). Fukui et al., in a retrospective study with a large number of patients, also confirmed that non-cancer-related mortality events in LUSC patients are significantly higher than in LUAD patients (43). Hence, LUSC patients often have comorbidities that could be one of the significant factors influencing the total OS. However, we believe that comorbidity may not be the sole reason for this OS difference. Unlike most studies conducted on surgical treatments, the patients selected for MWA treatment in this study mostly had indications that rendered them unsuitable for surgery. The patients’ baseline conditions were relatively similar, and the bias brought about by comorbidities can be disregarded. Our study indicated a worse prognosis for LUSC patients, suggesting that there may be more potential explanations for this phenomenon. Wang et al. conducted a retrospective study on Chinese patients and found that platelet levels and systemic inflammation indices were significantly higher in LUSC patients than in LUAD patients, which may contribute to the tumor’s development (44). Additionally, differences in gene expression between LUSC and LUAD involve several ontological subgroups, including cell proliferation, DNA replication, and cytoskeleton. Whole-genome analysis suggests significant structural differences in genes between the two. Research has shown that even the same expressed genes may have opposite effects in the signaling networks of LUAD and LUSC (45). Therefore, we believe that while the results show LUSC to be one of the risk factors for a worse prognosis, the impact of different histological types on prognosis awaits further explanation from pathological research.

Our study had several limitations. Firstly, despite being rooted in real-world data, the inherent nature of a single-center retrospective analysis introduces potential bias into the results, necessitating further validation through multi-center prospective studies. Secondly, our study did not incorporate repeated measurements at various time points to observe the dynamic changes of these biomarkers. This aspect will be addressed in our upcoming prospective registry study.

## 5 Conclusions

Elevated preoperative SIRI is an independent risk factor for poor prognosis in lung carcinoma patients undergoing MWA. Using nomograms based on SIRI to predict the long-term prognosis of these patients can assist clinical physicians in screening high-risk patients and formulating supportive treatments and personalized strategies.

## Data availability statement

The raw data supporting the conclusions of this article will be made available by the authors, without undue reservation.

## Ethics statement

The study was conducted in accordance with the Declaration of Helsinki (as revised in 2013) and was approved by the Ethics Committee of Beijing Chaoyang Hospital (2023-D.-471).

## Author contributions

JW: Conceptualization, Formal analysis, Investigation, Methodology, Project administration, Supervision, Visualization, Writing – original draft, Writing – review & editing. S-PC: Conceptualization, Formal analysis, Investigation, Methodology, Project administration, Software, Supervision, Validation, Writing – original draft, Writing – review & editing. QZ: Conceptualization, Data curation, Formal analysis, Investigation, Methodology, Writing – original draft. YG: Investigation, Methodology, Validation, Writing – original draft. YJ: Investigation, Methodology, Validation, Writing – original draft. YL: Investigation, Methodology, Writing – review & editing. J-BM: Data curation, Investigation, Project administration, Writing – original draft. Y-LF: Conceptualization, Data curation, Formal analysis, Resources, Visualization, Writing – original draft, Writing – review & editing. BH: Formal analysis, Funding acquisition, Investigation, Methodology, Resources, Software, Supervision, Validation, Writing – original draft, Writing – review & editing.
