# Supplementary material for: Preoperative systemic immune-inflammation index-based nomogram for lung carcinoma following microwave ablation -a real world single center study
Source: Front Oncol. 2024 Mar 20;14:1305262. doi: 10.3389/fonc.2024.1305262 (PMC10987766; doi:10.3389/fonc.2024.1305262)
Supplement: Supplementary file 1 [file DataSheet_1.docx]

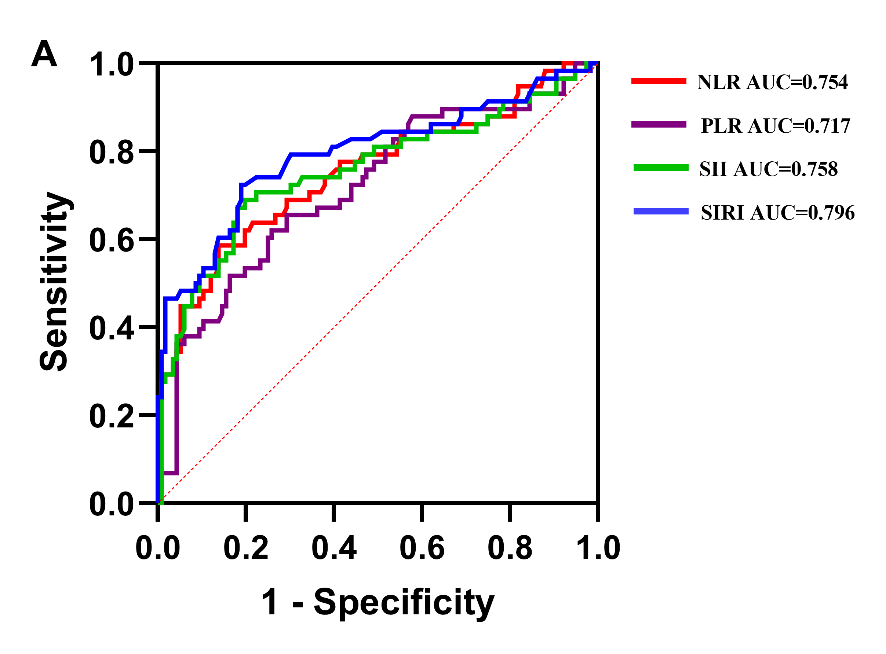

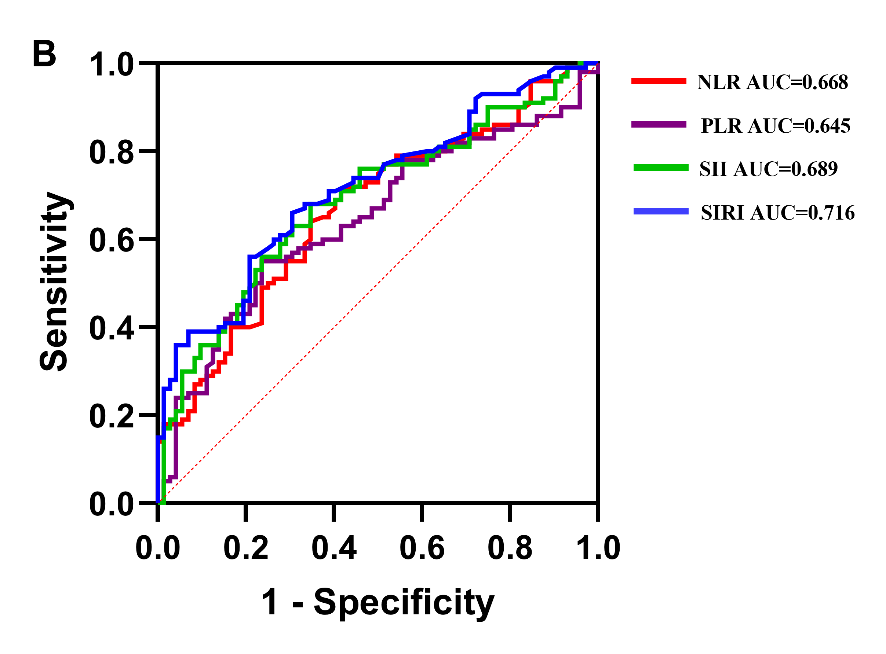


**Supplementary Figure 1** The ROC explores the value of inflammatory index in predicting the long-term prognosis in lung carcinoma patients who underwent MWA: (A) The value of ROC in predicting overall survival; (B) The value of ROC in predicting disease-free survival.
